# Supplementary material for: Curated and harmonised transcriptomics datasets of interstitial lung diseases
Source: Data Brief. 2025 Oct 14;63:112139. doi: 10.1016/j.dib.2025.112139 (PMC12581653; doi:10.1016/j.dib.2025.112139)
Supplement: Supplementary file 1 [file mmc1.zip › Supplementary_material/RNA-seq/fastQC reports/GSE184316/SRR15926784_fastqc.html]

SRR15926784.fastq.gz FastQC Report 

FastQC Report

Mon 5 Feb 2024  
SRR15926784.fastq.gz

## Summary

- Basic Statistics
- Per base sequence quality
- Per sequence quality scores
- Per base sequence content
- Per sequence GC content
- Per base N content
- Sequence Length Distribution
- Sequence Duplication Levels
- Overrepresented sequences
- Adapter Content

## Basic Statistics

| Measure | Value |
| --- | --- |
| Filename | SRR15926784.fastq.gz |
| File type | Conventional base calls |
| Encoding | Sanger / Illumina 1.9 |
| Total Sequences | 7569550 |
| Sequences flagged as poor quality | 0 |
| Sequence length | 25-378 |
| %GC | 51 |

## Per base sequence quality

## Per sequence quality scores

## Per base sequence content

## Per sequence GC content

## Per base N content

## Sequence Length Distribution

## Sequence Duplication Levels

## Overrepresented sequences

| Sequence | Count | Percentage | Possible Source |
| --- | --- | --- | --- |
| CAGTGGGGCCACGAGCTGAGTGCGTCCTGTCACTCCACTCCCATGTCCCT | 73120 | 0.9659755203413677 | No Hit |
| GAAAGGTGTGAGTTGGCCAGAACTCTGAAAAGATTGGGAATGGATGGCTA | 51184 | 0.6761828642389573 | No Hit |
| TGATATGGTTCCTGGCAAGCCCATGTGTGTTGAGAGCTTCTCAGACTATC | 36258 | 0.4789980910357947 | No Hit |
| TGATGACACCCACCGCAACTGTCTGTCTCATATCACGAACAGCAAAGCGA | 35886 | 0.4740836641544081 | No Hit |
| AGGCTACACACTGAGGACTCTGTTCCTCCCCTTTCCGCCTAGGGGAAAGT | 35360 | 0.4671347702307271 | No Hit |
| GTAACCACTCTCCCATTTGGCCAAACACATCCAGTTTGCTAGGCTGATTC | 35022 | 0.4626695113976393 | No Hit |
| GTAACCACTCTCCCATTGGCCAAACACATCCAGTTTGCTAGGCTGATTCC | 31523 | 0.4164448348977152 | No Hit |
| CACCTCCCCGAAGCTCAGGGAGAGCCCTGTTAGGGCCGCCTCTGGCCCTA | 31390 | 0.41468779517936993 | No Hit |
| GTCCAGGGTGGGCATTGTCAGGGAAGCTGCAGAGTTATTGAACCACTTGG | 29961 | 0.3958095263258714 | No Hit |
| CAAGGGTGACACCATTCAGCTCTACCTGGGGGCCAAGTTGTTGGACTCAC | 27095 | 0.3579473020192746 | No Hit |
| CACCTCCCCGAAACTCAGGGAGAGCCCTGTTAGGGCCGCCTCTGGCCCTA | 26203 | 0.3461632461639067 | No Hit |
| GGGAAGCGGGGAATGTCTACGTGCGTATGCACGTGGCACTCTCTGCCCGA | 25764 | 0.3403636940108725 | No Hit |
| CATGGCATTTGAATCATTTGAATCACTGTCATCTTCTTGTTCACTTCCTG | 22327 | 0.29495808865784623 | No Hit |
| CCCTGGCTGCTACAGGAGAATAGCAGACAGGTATAGTTAAGAAACAACTT | 20174 | 0.2665151825405737 | No Hit |
| AGCTGCTAAGTGCTGTGTTGTCGTTCCCCCTGCTTAAAATAAAGTTGTTT | 19741 | 0.2607948953372393 | No Hit |
| TATCTTCTTATTTATTTTCTGCCACTGAATTCTAGATTACCAGAGTGGTT | 19042 | 0.2515605286972145 | No Hit |
| GGCAATGAGCGGTTCCGCTGCCCTGAGGCACTCTTCCAGCCTTCCTTCCT | 18907 | 0.24977706732896934 | No Hit |
| GTCCACGTCACACTTCATGATGGAGTTGAAGGTAGTTTCGTGGATGCCAC | 17126 | 0.22624858809308346 | No Hit |
| CAAGGGGGCAGACCACACAGACCTCCCCAGGGACAGCCTCCCCAGTAATC | 17010 | 0.22471613239888766 | No Hit |
| TTCAGCCCTGATCAAGACATGAGGGAGGCAGGGGCTCAGCTGAAGAAGCT | 15749 | 0.20805728213698305 | No Hit |
| CTTTGAAAGGTGTGAGTTGGCCAGAACTCTGAAAAGATTGGGAATGGATG | 15156 | 0.2002232629416544 | No Hit |
| TTACAAAAGCAGAATAAAAGCGAAAAGAAATGAAAATGTTACACTACATT | 14905 | 0.1969073458792134 | No Hit |
| ACAAGAAGTACATCAAAGATTACATGAAATCAATCAAAGGGAAACTTGAA | 14752 | 0.19488608966186893 | No Hit |
| CACCATGAAGTCCAGCGGCCTCTTCCCCTTCCTGGTGCTGCTTGCCCTGG | 14337 | 0.18940359730763387 | No Hit |
| GTCAGCCTGGAGGTCAGCCAGGGTCCTGGGCTCCTAAATGACACTAAAGT | 13497 | 0.1783065043496641 | No Hit |
| CAGCCTGCAGGAAGCATCATCATTCTTCCGGAGGGACAGACCAGCAAGAC | 12371 | 0.16343111545600464 | No Hit |
| CAATCCACTTGATCCCAACTCATCTCTCATTTATTTCGGCTTCTTTTATT | 12207 | 0.16126454016421057 | No Hit |
| CGAGCCACAGCTCTGCCAGTACCCCAAGACTCAGCACTAGTCTGATGACC | 12156 | 0.1605907880917624 | No Hit |
| AGCTCGTGTGTTGTAACCACTCTCCCATTGGCCAAACACATCCAGTTTGC | 11689 | 0.1544213328401292 | No Hit |
| CAGCCTGCAGGAAGCATCATCATTCTTCCAGAGGGACAGACCAGCAAGAC | 11344 | 0.14986359823239162 | No Hit |
| TCTCAGTCTTGAAAGGGCAGTTGATGGTCACCGTTCTGCCCAGGTCCACT | 11002 | 0.14534549609950392 | No Hit |
| GTTCTGCAGCCCCTGTCATAAAAGGTTTTACTCTTTCTGGTCTCTGTTCT | 10743 | 0.14192389243746326 | No Hit |
| CAATGGGGTACTTCAGGGTCAGGATGCCACGCTTGCTCTGGGCCTCGTCG | 10314 | 0.13625644853392868 | No Hit |
| AGCTCGTGTGTTGTAACCACTCTCCCATTTGGCCAAACACATCCAGTTTG | 10306 | 0.13615076193432898 | No Hit |
| CGAAGCGGTTGTACTTGCGGATGTAGTGCAGATAGTCTCGGCGGATGACA | 9917 | 0.131011751028793 | No Hit |
| CCAGTCTCGGCTCAGCCAAACACTGTCAGGGCCCCCAGCAGGGCCTTCAG | 9788 | 0.12930755461024765 | No Hit |
| CTCCACCTACGGCGAGGGCGAGAGCGGCCCCATGGGCAACATCATGATCG | 9764 | 0.1289904948114485 | No Hit |
| CAGCAGTTTACACCTGCTAGGGTGTTCAAAGGTCAGTGCTATAGAAATTC | 9655 | 0.12755051489190242 | No Hit |
| GTCTCAACATCTGTGTTGGGGAGAGTGGAGACAGACTGACGCGAGCAGCC | 9642 | 0.1273787741675529 | No Hit |
| AGAGGGCTCCCAGAAGTGTGTGGCTGAGCTGGGTCCCCAGGCCGTGGGGG | 9598 | 0.12679749786975447 | No Hit |
| TAGGAGGACAGACTCCAGCTTTGAAGGACTTTCCAGAGCCTTCCACAGCC | 9411 | 0.1243270736041112 | No Hit |
| CTCTCTTTCTGGCCTGGAGGCTATCCAGCGTACTCCAAAGATTCAGGTTT | 9317 | 0.1230852560588146 | No Hit |
| GGCAATGAGCGGTTCCGGTGTCCGGAGGCGCTGTTCCAGCCTTCCTTCCT | 9026 | 0.11924090599837509 | No Hit |
| CTCCGCCGCCTCCGCCGCAGACGCCGCCGCGATGCGCTACGTCGCCTCCT | 8740 | 0.11546261006268536 | No Hit |
| GTCCACGTCACACTTCATGATGGAGTTGAAGGTGGTCTCGTGGATGCCGC | 8711 | 0.1150794961391364 | No Hit |
| CAGGGTGAAGGCCCACCCGTGGAGGCCAGACCCAAAGCCCACGGTACCGA | 8633 | 0.11404905179303923 | No Hit |
| GTGTCCATTCGAGGGCGGATCCTCTCTGGCGTGGTGACCAAGATGAAGAT | 8591 | 0.11349419714514074 | No Hit |
| TTTTCCATGAGCTTAATGATGCTTTCTCTGGGCTTTTGGGGGAGGGTGTC | 8400 | 0.1109709295796976 | No Hit |
| CTGGAAGACGTACCTTTGTTGGAAGACGTACCTTTGTTGGAAGACGTACC | 8070 | 0.1066113573462095 | No Hit |
| GAAACCCAGACACATAGCAATTCAGGAAATTTGACTTTCCATTCTCTGCT | 8050 | 0.10634714084721021 | No Hit |
| CGATCAGCACGGGCTCGTACTCTGCCACAAACTGATCACACTGCTTCTGG | 7684 | 0.10151197891552338 | No Hit |

## Adapter Content

Produced by FastQC (version 0.11.7)
